# High‐intensity interval training has a greater effect on metabolic profiles than the dietary composition in male rats

**DOI:** 10.14814/phy2.70802

**Published:** 2026-08-09

**Authors:** Asiyeh Taji Tabas, Marziyeh Saghebjoo, Reza Ghahremani, Mehdi Hedayati

**Affiliations:** ^1^ Department of Sport Sciences, Faculty of Sport Sciences University of Birjand Birjand Iran; ^2^ Cellular and Molecular Endocrine Research Center, Research Institute for Endocrine Molecular Biology, Research Institute for Endocrine Sciences Shahid Beheshti University of Medical Sciences Tehran Iran

**Keywords:** high‐fat diet, high‐intensity interval training, metabolomics, rats, skeletal muscles

## Abstract

Exercise and dietary factors play an important role in shaping the metabolic profile of skeletal muscle. This study compared the effects of 12 weeks of high‐intensity interval training (HIIT) with a normal diet (ND) or a high‐fat diet (HFD) on food and energy intake, as well as the metabolic profile of the gastrocnemius muscle in male rats. Thirty‐two Wistar rats were assigned to four groups (*n* = 8): normal diet control (NDC), high‐fat diet control (HFDC), HIIT+ND, and HIIT+HFD. Our findings demonstrated that HIIT decreased gastrocnemius muscle mass and the levels of beta‐alanine, glycine, serine, tyrosine, and fructose 6‐phosphate, while increasing glutamic acid levels. The HIIT+ND group exhibited significantly lower lactic acid levels than the other groups and lower threonine levels than the NDC and HFDC groups. Food and energy intake increased to a lesser extent in the HFDC and HIIT+HFD groups than in the ND and HIIT+ND groups. These findings indicate that HIIT exerts a predominant effect on metabolic regulation and can attenuate metabolic disturbances independently of diet. However, HIIT under a ND significantly affects certain glycolytic metabolites, including lactic acid.

## Graphical abstract

High‐intensity interval training (HIIT) induces skeletal muscle metabolic adaptations by modulating amino acid‐related pathways largely independent of dietary fat intake, while under a normal diet it also alters specific glycolytic metabolites, including lactic acid.

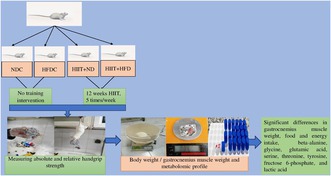

## 1 INTRODUCTION

High‐fat diets (HFDs) and sedentary lifestyles are major public health concerns that significantly increase the risk of obesity, diabetes, and cardiovascular disease. These factors contribute to skeletal muscle atrophy, reduced muscle fiber diameter, and decreased muscle mass, often accompanied by increased fat tissue accumulation (Ferretti et al., 2018; Lee et al., 2021). Prolonged exposure to HFD and excessive lipid accumulation impairs skeletal muscle function in rodents, particularly by reducing contractile force in fast‐twitch muscle fibers and leading to decreased muscle strength (Andrich et al., 2018). Furthermore, high‐fat and high‐sugar diets exacerbate muscle atrophy, promote protein breakdown, induce inflammation in peripheral tissues, and impair glucose transport (Rasool et al., 2018). High‐fat/high‐sucrose diets can also cause structural and inflammatory alterations in skeletal muscle, accelerating the progression of sarcopenia by disrupting postprandial stimulation of muscle protein synthesis (Wada et al., 2023). HFDs containing 40%–45% kcal from fat have been shown to increase intramuscular fat deposition, tissue fibrosis, and inflammatory cell infiltration while attenuating muscle hypertrophy (Collins et al., 2016; DE Sousa et al., 2021). In addition to these anatomical and functional changes, HFDs have a profound impact on skeletal muscle metabolomics. Metabolomics enables comprehensive profiling of small molecule metabolites in cells, tissues, and biofluids, offering valuable insights into muscle metabolism and the pathways involved in hypertrophy, fat deposition, and protein degradation (Cônsolo et al., 2020; Goldansaz et al., 2017). HFDs have been reported to alter the amino acid profile, carbohydrate and lipid metabolism, as well as purine and pyrimidine metabolites, and protein kinase B phosphorylation in skeletal muscle (Liu et al., 2013). For example, six weeks of HFD feeding increases the levels of alanine, glucose, glucose‐6‐phosphate, and glycerate in the gastrocnemius muscle of obese rats (Liu et al., 2013). Overall, skeletal muscle exhibits remarkable plasticity and can adapt to a wide range of environmental stimuli, including dietary intake and physical activity (Lahiri et al., 2019).

Exercise training is a well‐established intervention for counteracting the detrimental effects of HFD on skeletal muscle. Recent evidence indicates that exercise training can significantly influence the metabolomic profile of skeletal muscle in HFD‐fed animals, primarily by modulating amino acid metabolism (Han et al., 2023). Skeletal muscle plays a central role in exercise performance, energy expenditure, and the utilization of glucose and amino acids, processes that are regulated by various metabolites (Kamei et al., 2020). Eight weeks of exercise training to treadmill running or wheel running in obese rats have been shown to significantly reduce amino acids, carnitines, and fatty acids, effectively reversing the metabolic alterations induced by an HFD (Han et al., 2023). Similarly, rats with high intrinsic running capacity display a more favorable muscle metabolic profile, with higher levels of glutamine and glutamate and lower lactate concentrations, reflecting more efficient glucose oxidation (Shi et al., 2018). These findings underscore the capacity of skeletal muscle to undergo physiological and biochemical adaptations in response to physical activity, thereby enhancing whole‐body metabolic health (Lee & Farrar, 2003; Watt et al., 2021).

Despite these benefits, lack of time remains a common barrier to regular physical activity. High‐intensity interval training (HIIT) is an effective and time‐efficient exercise modality that has been shown to improve energy metabolism and body composition (Fayh et al., 2018; Tsirigkakis et al., 2021). However, the combined effects of HFD and regular exercise, particularly HIIT, on skeletal muscle metabolism remain incompletely understood. Investigating the metabolite profiles resulting from the interaction between HFD and exercise training may provide a deeper understanding of the underlying metabolic mechanisms and help identify new therapeutic targets for skeletal muscle health.

Given that fast‐twitch muscle fibers are more susceptible to atrophy and metabolic disturbances following HFD exposure, the gastrocnemius muscle was selected for investigation in this study. Previous research has demonstrated weight loss and reduced mechanistic target of rapamycin gene expression in this muscle after HFD feeding (Renzini et al., 2021; Yan et al., 2021). Additionally, regular exercise influences the biological regulation of body weight and food intake in a sex‐specific manner, with distinct mechanisms underlying eating behavior and metabolic control in males and females (Foright et al., 2020; Ruoppolo et al., 2018). To avoid the confounding effects of hormonal cycles, only male rats were used in the present study. Animal models, particularly rats, are widely utilized in biomedical research due to their physiological similarities to humans in metabolic and exercise responses (Goutianos et al., 2015). Rats have been shown to reflect human responses to exercise in blood parameters adequately and are a justified model for studying diet‐induced obesity and metabolic dysregulation (Goutianos et al., 2015; Sadie‐Van Gijsen & Kotzé‐Hörstmann, 2023).

Therefore, the present study aimed to compare the effects of 12 weeks of HIIT combined with either a normal diet (ND) or an HFD on food and energy intake, gastrocnemius muscle mass, and the metabolomic profile of the gastrocnemius muscle in male Wistar rats. We hypothesized that HIIT combined with an ND would induce more favorable metabolic and anatomical adaptations in skeletal muscle compared to HIIT combined with an HFD.

## 2 MATERIALS AND METHODS

### 2.1 Animals and experimental design

**FIGURE 1:**
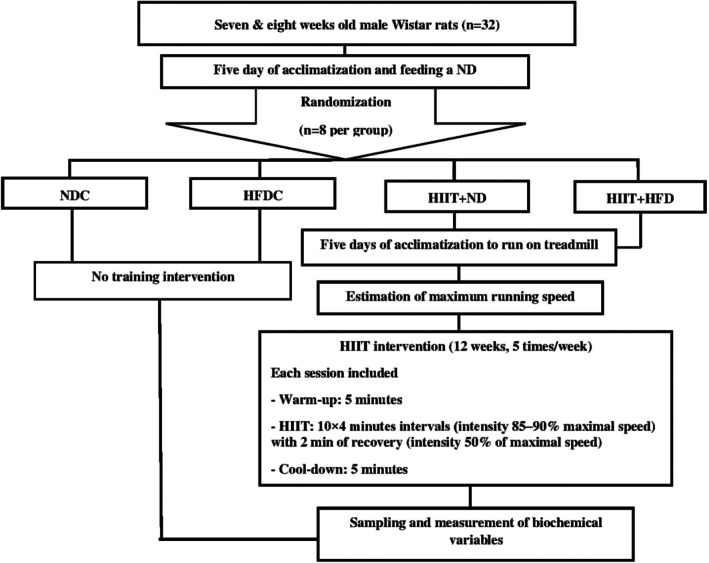
The study flowchart and HIIT protocol. HFD, High‐fat diet; HFDC, High‐fat diet control; HIIT, High‐intensity interval training; ND, Normal diet; NDC, Normal diet control.

In this study, 32 male Wistar rats (7‐8‐week‐old, weighing 150–200 g) were purchased from the Pasteur Institute of Karaj, Iran, and then transferred to the animal house of the University of Birjand. The animals were kept in standard polycarbonate cages under controlled environmental conditions. The conditions included a 12‐h light/dark cycle, with a temperature maintained between 22°C and 24°C and a relative humidity ranging from 20% to 30%. After 5 days of acclimatization and feeding an ND, rats were randomly divided into four equal groups (*n* = 8 per group, three cages to keep rats of each group, two cages contained three rats, one cage contained two rats): normal diet control (NDC), high‐fat diet control (HFDC), HIIT+ ND, and HIIT+HFD (Figure 1). The limited number of cages and laboratory space made it impossible to house one rat per cage. MedCalc Ver Software. 19.8 was used to determine the sample size. Since the amino acid tyrosine undergoes the least changes among metabolites, especially amino acids in rats, it requires the largest sample size. A sample size of six rats per group was calculated with a power of 90% and a type I error of 0.05 based on changes in mean tyrosine levels, but due to the risk of sample loss, the number of rats per group was set at eight. From the time of familiarization until the end of the training intervention, all rats were given free access to water (tap water in 500 mL bottles) and food. All the procedures performed in this study adhered to ethical standards and were performed according to guidelines from the Research Ethics Committees of the University of Birjand, Birjand, Iran (Approval ID: IR.BIRJAND.REC.1400.011).

### 2.2 Composition of the diets and measurement of food intake

The rats' food was purchased as pellets from the Royan Institute for Animal Biotechnology, Isfahan, Iran. The HFD contained 43% kcal from carbohydrates, 40% kcal from fat, and 17% kcal from protein (Gopalan et al., 2021), with an energy density of 4.7 kcal per gram; and the ND contained 64% kcal from carbohydrates, 16% kcal from fat, and 20% kcal from protein (Pirman et al., 2021) and had an energy density of 3.4 kcal per gram. The composition of the diets is presented in Table 1. In addition, measuring food intake required subtracting the weight of food remaining after 24 h from the weight of food initially placed. The determination of the total average daily energy intake involved multiplying the average daily food intake (in grams) by the energy obtained per gram of food. The HIIT+HFD and HFDC groups switched to HFD at the initiation of the HIIT protocol.

**TABLE 1:** The composition of the experimental diets.

Ingredients	Normal diet (g/kg)	High‐fat diet (g/kg)
Casein, Lactic, 30 Mesh	200	195
Methionine, DL	‐	3
Cysteine, L	3	‐
Dextrose, Monohydrate	150	‐
Sucrose, Fine granulated	4	350
Lodex 10	110	100
Starch, Corn	381	50
Solka Floc, FCC200	75	50
Raftiline HP	25	‐
Margarine	‐	200
Corn oil	‐	10
Soybean oil, USP	70	‐
Mineral S10001A	‐	17.5
Mineral S10026B	50	‐
Calcium phosphate, Dibasic	‐	17.5
Calcium carbonate, Light, USP	‐	4
Choline bitartrate	2	2
Vitamin V10001C	1	1
Ethoxyquin	‐	0.04
Cholesterol, NF	‐	1.5
Total	1071	1001.54

### 2.3 HIIT protocol

The acclimatization program on the treadmill (0° slope) in the HIIT+HFD and HIIT+ND groups for 5 days (one session per day) is shown in Table 2 (Groussard et al., 2019; Qin et al., 2020).

**TABLE 2:** Treadmill acclimation protocol.

Days	Duration and speed of walking/running on the treadmill
1	10 min at 12 m·min−1
2	20 min at 12 m·min−1
3	30 min at 12 m·min−1
4	10 min at 12 m·min−1 + 5 min at 18 m·min−1
5	(5 min at 10 m·min−1 + 4 min at 18 m·min−1) × 2

**FIGURE 2:**
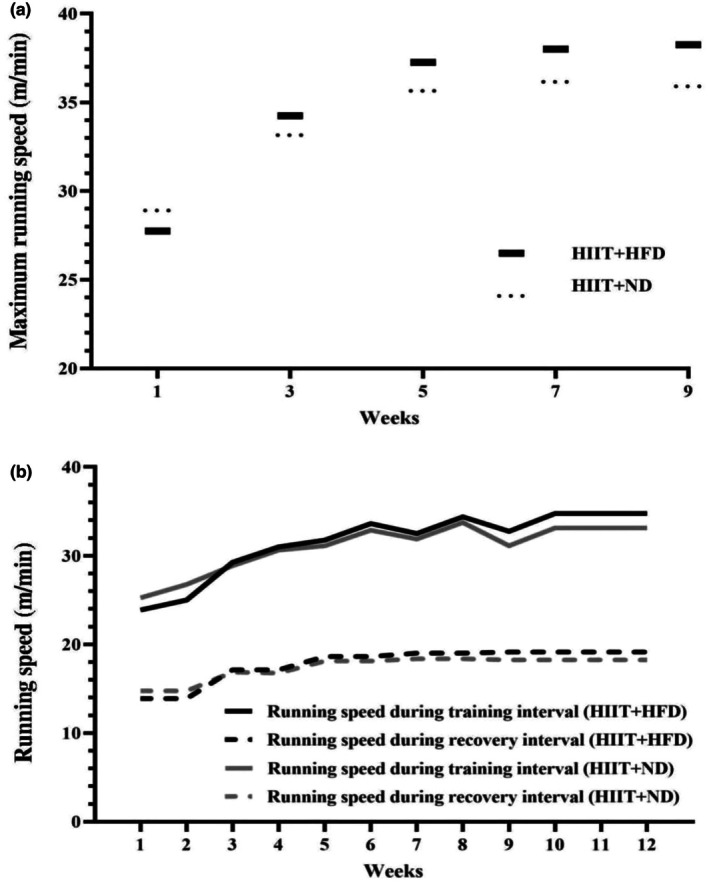
Changes in maximum running speed (a) and running speed (b) during HIIT protocol; HFD, High‐fat diet; HIIT, High‐intensity interval training; ND, Normal diet.

Forty‐eight hours after the last acclimatization session, the maximum running speed was estimated using a treadmill (slope of 25°). For this purpose, the rats were placed on a treadmill and given 5 min to adapt at a speed of 6 m/min. Subsequently, the speed was gradually increased by 2 m/min every 2 min until the rats were unable or unwilling to continue for 15 s (Batista Jr et al., 2010; Chavanelle et al., 2017, Khalafi et al., 2020). The running speed of the last stage was defined as the maximal speed (Khalafi et al., 2020). In addition, the maximum running speed was measured at the beginning of every 2 weeks from the 12‐week protocol (except for the last 2 weeks) (Figure 2a), and the new training intensity was determined based on it (Khalafi et al., 2020).

The HIIT protocol involved treadmill running for 60 min per session, conducted 5 days a week for 12 weeks. Each session of HIIT comprised 10 × 4‐min intervals at 85%–90% maximal speed with 2 min of recovery between each interval at 50% maximal speed (Figure 2b). Interval speed was gradually increased over the 10 weeks based on the newly obtained maximum speed and remained constant during the final 2 weeks. The warm‐up and cool‐down were included at the beginning and end of each training session, respectively, comprising 5 min of running at the intensity of 45%–50% of maximal speed (Khalafi et al., 2020) (Figure 1). To equalize the stress response to treadmill noise across all groups, control group cages were positioned near the treadmill during the HIIT sessions, without access to food or water, like the training groups.

### 2.4 Measuring absolute and relative handgrip strength

According to laboratory practice guidelines, individuals refrain from exercise for at least 48 h before sampling (Đerek et al., 2024). This is because the physiological effects of resistance exercise can persist for up to 48 h, as biochemical markers gradually return to baseline (Timón et al., 2019). Therefore, a 48‐h interval was maintained between muscle strength testing and tissue sampling, with muscle strength testing conducted immediately before the final training session as described below:

Muscle strength was assessed using a weight test consisting of a custom‐made wire plate equipped with metal rings to catch the rats. Rats were acclimated to the apparatus before testing. To measure muscle strength, the rat was held by the base of its tail and lowered to grasp the first weight, which was placed on the laboratory bench. Immediately after the rat holds the first weight (70 grams) with its paws, the experimenter, holding the rat's tail, raises the rat to break the contact between the wire plate and the bench. A hold of 3 s is the criterion. If the rat released the weight in under 3 s, a resting period of approximately 10 s was implemented before attempting the weight again. After three unsuccessful attempts, the trial was ended, and the rat was assigned the maximum time/weight achieved. By multiplying the number of links in the longest chain held continuously for 3 s by the duration (in seconds) it was maintained, the final total score was calculated. For example, a rat successfully lifting a 5‐link weight for 3 s, but unable to lift a 6‐link weight, was assigned a score of (5 × 3) = 15. If it held the 6‐link weight for 1 s, it scores (5 × 3) + (1) = 16 (Deacon, 2013). The determination of relative handgrip strength was achieved through the division of muscle strength by the body weight of each rat (Ahmadabadi et al., 2020).

### 2.5 Sampling and measurement of biochemical variables

To minimize potential interference from the acute effects of the final running session, the animals were anesthetized using ketamine (100 mg/kg) and xylazine (10 mg/kg) 48 h after the last training session (Shakoor et al., 2023; Szczerbinski et al., 2021), following a 12‐h overnight fast. The gastrocnemius muscle of the right limb was harvested immediately by surgery, washed in normal saline, and weighed using an automatic balance with a sensitivity of 0.01 g, manufactured by Sartorius Company, Germany. Then, each sample was placed in a gated microtube and immediately immersed in liquid nitrogen (−196°C) for sudden freezing. Finally, the samples were transferred to the laboratory and were stored at −80°C for subsequent gas chromatography–mass spectrometry (GC–MS) analysis.

### 2.6 Measurement of body weight and gastrocnemius muscle mass

Body weight was measured at the beginning and the end of the protocol training using an automatic balance (sensitivity: 0.1 g, Kern Company, Germany). Also, after tissue removal, the gastrocnemius muscle mass of the right limb was recorded in each rat.

### 2.7 Tissue sample preparation for GC–MS analysis

In a comprehensive study on metabolite profiling of skeletal muscle samples, GC–MS was employed as the analytical technique. Each muscle sample, weighing 100 mg, was subjected to homogenization in 800 μL of a chilled chloroform/methanol/water mixture (2:3:1, v/v/v). Subsequently, the homogenized samples underwent centrifugation at a speed of 14,200 g and a temperature of 4°C for 14 min. This process led to the separation of the supernatant, which was then meticulously transferred to a fresh 1.5 mL microcentrifuge tube. The remaining precipitate was resuspended in 400 μL of the chilled solvent mixture. The entire supernatant solution was then evenly distributed among three microtubes and stored at −80°C until analysis.

### 2.8 Metabolite extraction and derivatization

To effectively extract metabolites from the muscle samples, 1 mL of protein precipitant, comprising a mixture of methanol, water, and isopropanol in a 5:2:2 ratio (v/v/v), was added to 50 μL of the liquid sample held in 1.5 mL tubes. The tubes were vigorously shaken, cooled to 20°C for 20 min, and subsequently centrifuged at 14,000 rpm for 15 min at 4°C to eliminate the precipitated protein. The resulting supernatant was carefully collected and subjected to concentration to dryness using a vacuum centrifuge operated at 45°C for 3 h. To render the aldehyde and ketone functional groups more volatile and stable for subsequent GC–MS analysis, the dried samples were subjected to methoximation and trimethylsilylation. In this method, after adding 30 μL of a 20 mg/mL solution of methoxyamine hydrochloride in pyridine to the dried samples, they were subjected to heating at 60°C for 1 h on a thermal shaker. Next, 60 μL of N‐methyl‐N‐(trimethylsilyl) trifluoroacetamide (MSTFA) was introduced as a silylating agent. The samples were then thoroughly shaken and incubated for 20 min at 45°C.

### 2.9 Untargeted GC–MS analysis

Researchers employed an Agilent 5975C MSD/7890A GC system, equipped with an HP‐5 ms capillary column, to conduct an untargeted GC–MS analysis. They injected 1 mL of each derivatized sample using a 1:4 split ratio and a steady helium flow of 1 mL/min. The inlet, transfer line, and ion source temperatures were maintained at 280°C, 150°C, and 230°C, respectively. To minimize systematic bias, samples were analyzed in a randomized order. The chromatographic parameters were: (a) initial temperature: 60°C; (b) hold time: 1 min; (c) ramp rate: 10°C/min; (d) final temperature: 280°C; (e) hold time: 22 min. Under electron impact ionization (EI) conditions, mass spectra were collected within the m/z range of 50–600 with a 5.4‐min delay following solvent elution.

### 2.10 GC–MS data processing

Agilent Mass Hunter qualitative data analysis software was employed to process the raw GC–MS data, specifically for peak identification and mass spectrum deconvolution. Following that, the processed data was exported to CDF (NetCDF) format and subsequently converted to “abf” format for further analysis using MS‐DIAL software (v4.0) to annotate metabolites. Automated metabolite annotation was performed using the built‐in MS/MS reference libraries within MS‐Dial. Before statistical analysis, MS‐Dial generated a peak list for each sample, containing the mean retention time (RT), m/z values, MS/MS spectra, and peak intensity (height). Annotations generated by MS‐Dial against various spectral libraries (replib, mainlib, and Fiehn) with a 70% similarity threshold were subsequently validated using the NIST Mass Spectral Search program (version 2.0) for enhanced confidence. Finally, the MS‐Dial preprocessed data was subjected to the following post‐processing steps: (a) removal of all contaminating ions generated by the derivatization reagents from the original data set; (b) summation of the peak intensities of duplicate features originating from the same molecule (resultant from insufficient derivatization); and (c) normalization of metabolite peaks using the mTIC approach by intensity dividing of the metabolites by the product of the sum of all detected metabolites and the average mTIC.

### 2.11 Statistical analyses

Statistical analyses were performed using SPSS software version 24 employing a significance level of *p* ≤ 0.05. Data are presented as mean ± standard deviation. The Shapiro–Wilk test was used to examine the normal distribution of data. If the data conformed to a normal distribution (gastrocnemius muscle mass, aspartic acid, beta‐alanine, glycine, glutamic acid, 4‐hydroxyproline, lysine, phenylalanine, serine, tyrosine, fructose 6‐phosphate, glucose, lactic acid, palmitic acid, stearic acid, hypoxanthine, nicotinamide, and absolute and relative handgrip strength), a two‐way analysis of variance (ANOVA) was used to analyze the main effects of HIIT and diet, as well as their interaction. When there was a significant interaction effect between HIIT and diet, Tukey's post‐hoc test was performed. Since the Kruskal‐Wallis and the Mann–Whitney U tests are used when the data distribution is not normal, these tests were used to analyze the distributions of alanine, isoleucine, methionine, 3‐phosphoglycerate, threonine, and valine. The repeated measures ANOVA test was employed to compare the means of body weight and food and energy intake across the various groups, with the Bonferroni post‐hoc test. Also, the Pearson correlation coefficient was used to find probable associations among variables.

## 3 RESULTS

### 3.1 Body weight

**FIGURE 3:**
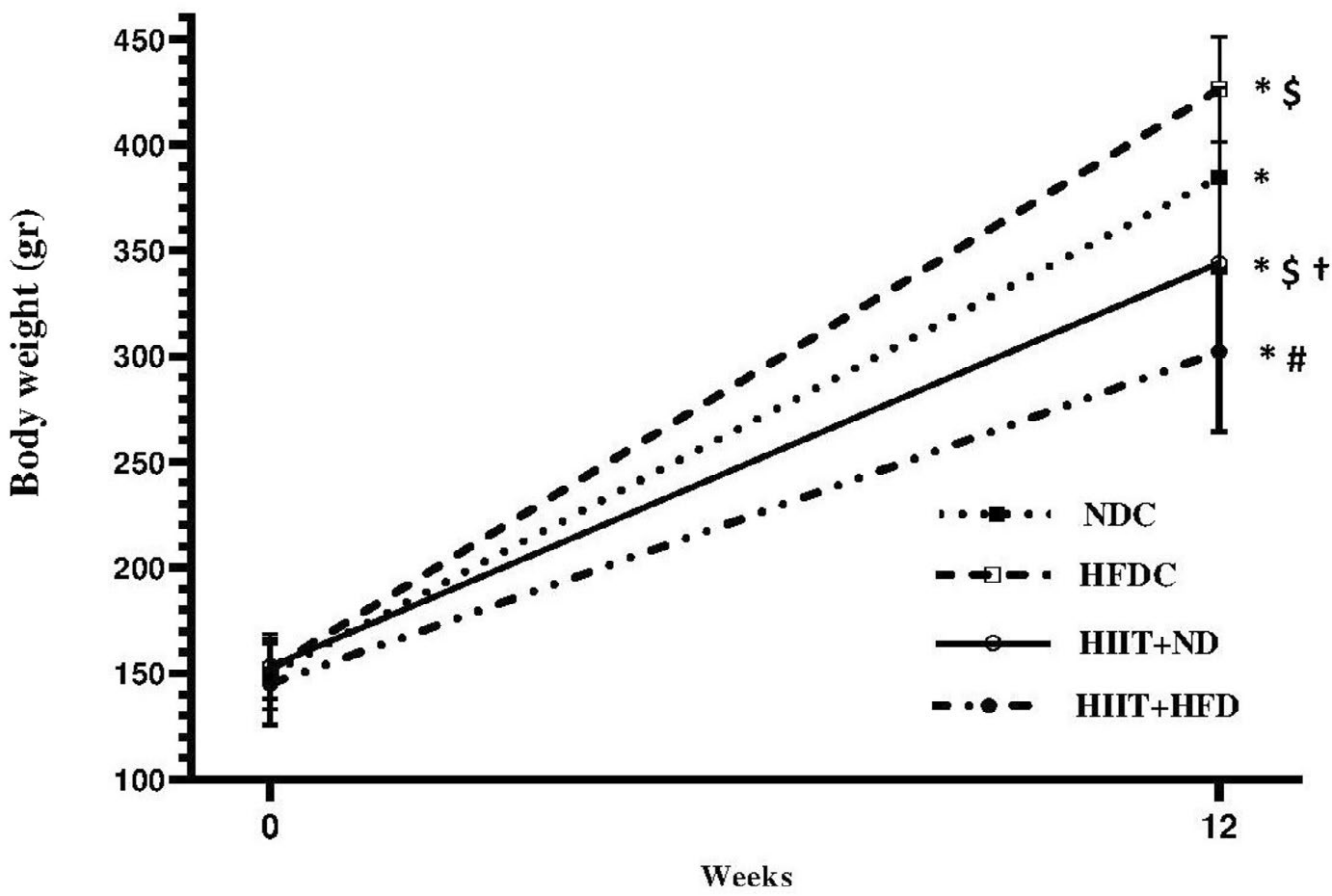
Change of body weight following 12 weeks of intervention; HFD, High‐fat diet; HFDC, High‐fat diet control; HIIT, High‐intensity interval training; ND, Normal diet; NDC, Normal diet control. Data are expressed as means±SD (*n* = 8) in each group. *Significant difference compared with the first week; ^#^significant difference compared with the other groups in the final week; ^$^significant difference compared with the NDC group in the final week, ^†^significant difference compared with the HFDC group in the final week.

The statistical analysis using repeated measures ANOVA showed a significant effect of time, group, and time × group interactions (*F*_[1,28]_ = 1372.81, *p* = 0.01; *F*_[3,28]_ = 10.93, *p* = 0.01; *F*_[3,28]_ = 19.53, *p* = 0.01; respectively). Body weight significantly increased in all groups compared to baseline (*p* = 0.001 for all groups). Based on pairwise comparisons at the end of the intervention, the body weight was significantly lower in the HIIT+HFD group than in the HIIT+ND (*p* = 0.03), HFDC (*p* = 0.001), and NDC (*p* = 0.001) groups, and in the HIIT+ND group was significantly lower than in the HFDC (*p* = 0.001) and NDC (*p* = 0.03) groups. In addition, the HFDC group exhibited a notably higher body weight compared to the NDC group (*p* = 0.03) (Figure 3).

### 3.2 Gastrocnemius muscle mass

**FIGURE 4:**
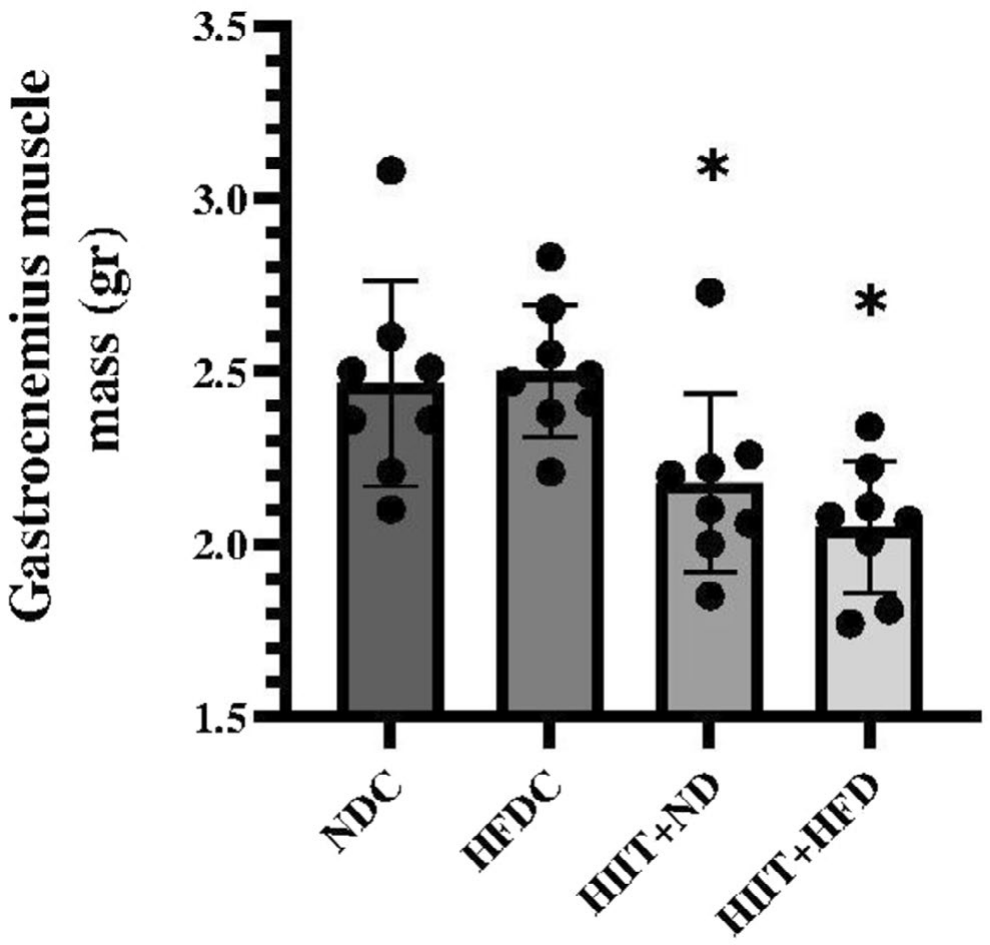
Changes in gastrocnemius muscle mass following 12 weeks of intervention. HFD, High‐fat diet; HFDC, High‐fat diet control; HIIT, High‐intensity interval training; ND, Normal diet; NDC, Normal diet control. Data are expressed as means±SD (*n* = 8) in each group. *significant difference for HIIT effect.

A two‐way ANOVA showed that HIIT significantly reduced the muscle mass (*F* = 19.10, *p* = 0.001). Furthermore, diet and the interaction of HIIT × diet did not have a significant effect on muscle mass (*F* = 0.27, *p* = 0.60 and *F* = 0.93, *p* = 0.34; respectively) (Figure 4).

### 3.3 Gastrocnemius muscle metabolites

Based on untargeted GC–MS analysis, twenty‐two metabolites in the gastrocnemius muscle were identified. These metabolites were mainly categorized as glycolytic metabolites, amino acids, fatty acids, and nucleotides.

#### 3.3.1 Glycolytic metabolites

**FIGURE 5:**
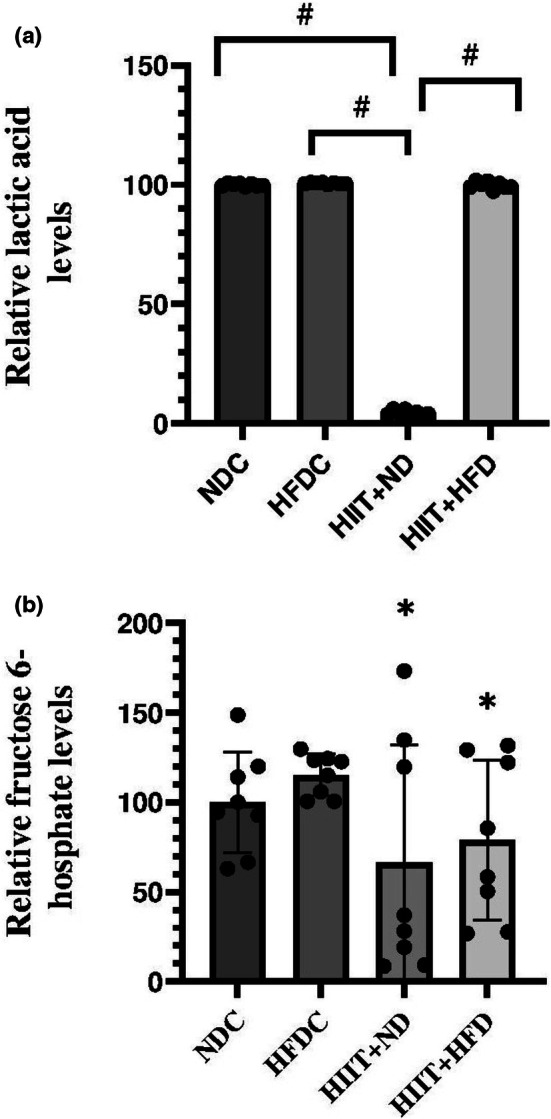
Changes in levels of lactic acid (a) and fructose 6‐phosphate (b) following 12 weeks of intervention; HFD, High‐fat diet; HFDC, High‐fat diet control; HIIT, High‐intensity interval training; ND, Normal diet; NDC, Normal diet control. Data are expressed as means±SD (*n* = 8) in each group. ^#^significant difference compared with the HIIT+ND group; *significant difference for HIIT effect.

Analysis of the changes of lactate and fructose 6‐phosphate levels, expressed relative to the NDC group baseline (set as 100). The two‐way ANOVA results showed significant effects of HIIT, diet, and the interaction of HIIT × diet on lactic acid levels (*F* = 17914.57, *p* = 0.001; *F* = 17780.90, *p* = 0.01; *F* = 17339.96, *p* = 0.001; respectively). Specifically, changes of nearly 100% in levels were demonstrated in the HIIT+ND group, with the lactic acid level in the HIIT+ND group being significantly lower than in the NDC, HFDC, and HIIT+HFD groups (*p* = 0.001 for all comparisons; Figure 5a). Also, HIIT led to a significant decrease in fructose 6‐phosphate levels (*F* = 5.44; *p* = 0.02, Figure 5b).

#### 3.3.2 Amino acids profile

**FIGURE 6:**
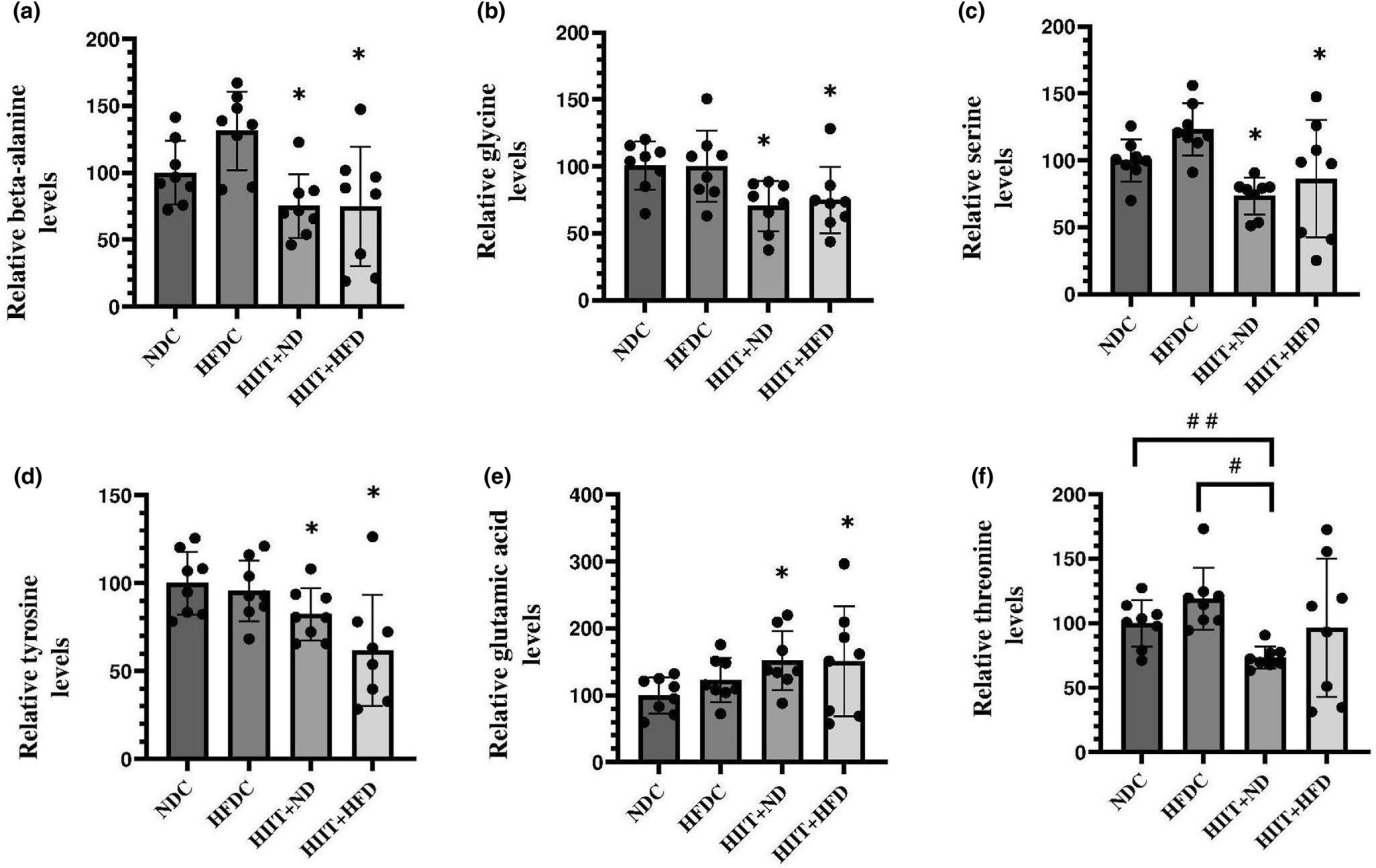
Changes in levels of beta‐alanine (a), glycine (b), serine (c), tyrosine (d), glutamic acid (e), and threonine (f) following 12 weeks of intervention; HFD, High‐fat diet; HFDC, High‐fat diet control; HIIT, High‐intensity interval training; ND, Normal diet; NDC, Normal diet control. Data are expressed as means±SD (*n* = 8) in each group. *significant difference for HIIT effect, ^#^significant difference compared with the HFDC group; ^# #^significant difference compared with the NDC group.

We analyzed the changes in amino acid levels, expressed relative to the NDC group baseline (set as 100). According to the results of two‐way ANOVA, HIIT had a significant reducing effect on the levels of beta‐alanine (*F* = 13.33; *p* = 0.001, Figure 6a), glycine (*F* = 12.15; *p* = 0.002, Figure 6b), serine (*F* = 11.79; *p* = 0.002, Figure 6c), and tyrosine (*F* = 11.47; *p* = 0.002, Figure 6d), and a significant increasing effect on glutamic acid level (*F* = 4.91; *p* = 0.03, Figure 6e). However, the diet and the interaction of HIIT × diet had no significant effects on these variables (*p* > 0.05). Finally, the Kruskal‐Wallis test showed a significant difference among groups for threonine (*χ* ^2^ = 11.29, *p* = 0.01). The results of Mann–Whitney *U* tests showed that the HIIT+ND group exhibited significantly lower threonine levels than the HFDC and NDC groups (*p* = 0.001 and *p* = 0.005, respectively; Figure 6f).

#### 3.3.3 Unchanged detected metabolites

The results shown in Table 3 show that no differences in other amino acids (branched‐chain amino acids (BCAAs: isoleucine, valine), alanine, aspartic acid, 4‐hydroxyproline, lysine, methionine, and phenylalanine), the metabolic profile of glycolysis (glucose and 3‐phosphoglycerate), fatty acids (palmitic acid and stearic acid), and nucleotides (hypoxanthine and nicotinamide) were observed among groups (*p* > 0.05).

**TABLE 3:** Changes in other metabolite levels in the gastrocnemius muscle of rats following the intervention.

Class	Metabolite	NDC	HFDC	HIIT+ND	HIIT+HFD	HIIT effect *F*(*p*)	Diet effect *F*(*p*)	Interaction *F*(*p*)	*χ* ^2^(*p*)
Amino acids (%)	Alanine	100.00 ± 11.47	101.80 ± 13.94	115.28 ± 31.31	96.51 ± 48.93	‐	‐	‐	2.62^B^ (0.45)
	Aspartic acid	100.00 ± 30.54	107.33 ± 21.41	109.91 ± 26.40	111.15 ± 74.24	0.19 (0.65)	0.07 (0.78)	0.03^A^ (0.84)	‐
	4‐hydroxyproline	100.00 ± 40.79	73.56 ± 33.76	95.68 ± 27.38	92.99 ± 24.64	0.43 (0.51)	1.63 (0.21)	1.08^A^ (0.30)	‐
	Isoleucine	100.00 ± 11.35	103.09 ± 12.62	98.79 ± 20.51	115.22 ± 57.93	‐	‐	‐	0.02^B^ (0.99)
	Lysine	100.00 ± 18.75	127.61 ± 34.33	129.48 ± 48.29	116.64 ± 54.84	0.39 (0.53)	0.25 (0.61)	1.90^A^ (0.17)	‐
	Methionine	100.00 ± 33.18	126.45 ± 26.70	95.38 ± 38.37	104.39 ± 63.49	‐	‐	‐	3.33^B^ (0.34)
	Phenylalanine	100.00 ± 13.63	106.55 ± 12.15	98.28 ± 18.84	96.32 ± 49.38	0.36 (0.55)	0.05 (0.81)	0.18^A^ (0.67)	‐
	Valine	100.00 ± 14.15	100.76 ± 16.17	86.56 ± 13.67	86.19 ± 51.69	‐	‐	‐	3.50^B^ (0.32)
Glycolysis (%)	Glucose	100.00 ± 5.40	99.95 ± 13.31	95.04 ± 12.6	98.55 ± 19.11	0.44 (0.51)	0.12 (0.72)	0.14^A^ (0.71)	‐
	3‐phosphoglycerate	100.00 ± 182.09	218.63 ± 246.47	216.18 ± 240.89	70.60 ± 56.06	‐	‐	‐	3.70^B^ (0.29)
Fatty acid (%)	Palmitic acid	100.00 ± 22.79	100.63 ± 20.99	118.11 ± 21.56	104.74 ± 14.03	2.43 (0.13)	0.80 (0.37)	0.96^A^ (0.33)	‐
	Stearic acid	100.00 ± 19.65	103.10 ± 22.60	94.42 ± 46.86	71.14 ± 29.76	2.83 (0.10)	0.81 (0.37)	1.39^A^ (0.24)	‐
Nucleotides (%)	Hypoxanthine	100.00 ± 28.39	92.25 ± 14.81	94.78 ± 21.09	117.11 ± 44.23	0.90 (0.35)	0.49 (0.48)	2.11^A^ (0.15)	‐
	Nicotinamide	100.00 ± 9.19	97.84 ± 11.81	100.85 ± 8.80	89.78 ± 27.59	0.38 (0.53)	1.31 (0.26)	0.59^A^ (0.44)	‐

*Note*: Data are represented as means ±SD; A indicates two‐way ANOVA, and B indicates Kruskal‐Wallis test values.

Abbreviations: HFD, high‐fat diet; HFDC, high‐fat diet control; HIIT, high‐intensity interval training; ND, normal diet; NDC, normal diet control.

### 3.4 Food and energy intake

**FIGURE 7:**
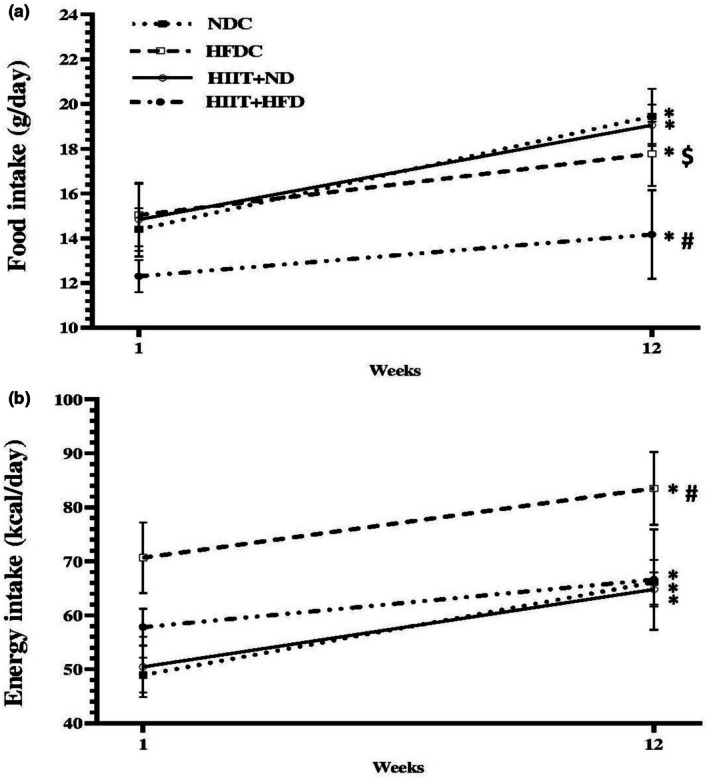
Changes in food intake (a) and energy intake (b) following 12 weeks of intervention; HFD, High‐fat diet; HFDC, High‐fat diet control; HIIT, High‐intensity interval training; ND, Normal diet; NDC, Normal diet control. Data are expressed as means±SD (*n* = 8) in each group. *Significant difference compared with the first week; ^#^significant difference compared with the other groups in the final week; ^$^significant difference compared with the NDC group in the final week.

Analysis of food intake using repeated measures ANOVA showed a significant effect of time, group, and time × group interactions (*F*_[1,28]_ = 83.27, *p* = 0.01; *F*_[3,28]_ = 38.82, *p* = 0.01; *F*_[3,28]_ = 3.56, *p* = 0.02; respectively). Compared to the first week, a significant increase in food intake was observed in all groups after the 12 weeks (*p* ≤ 0.05 for all groups), while the NDC group showed the highest food intake (35%), followed by the HIIT+ND (28%), HFDC (18%), and HIIT+HFD (15%) groups. Although, based on pairwise comparisons at the end of the intervention, food intake in the HIIT+HFD group was significantly lower than in the HIIT+ND, HFDC, and NDC groups (*p* = 0.001 for all comparisons) and in the HFDC group was significantly lower than in the NDC group (*p* = 0.02), the HFDC and HIIT+HFD groups showed a smaller increase in food intake at the end of intervention compared to the first week compared to the NDC and HIIT+ND groups (Figure 7a).

Analysis of energy intake using repeated measures ANOVA showed a significant effect of time, group, and time × group interactions (*F*_[1,28]_ = 74.06, *p* = 0.01; *F*_[3,28]_ = 52.75, *p* = 0.01; *F*_[3,28]_ = 1.28, *p* = 0.29; respectively). After the 12 weeks, energy intake significantly increased in all groups compared to the first week (*p* ≤ 0.05 for all groups), while the NDC group exhibited the greatest increase in energy intake (35%), followed by HIIT+ND (28%), HFDC (18%), and HIIT+HFD (15%) groups. Despite the higher energy intake in the HFDC group in the final week compared to the other groups (*p* = 0.001 for all comparisons), our data show that the HFDC and HIIT+HFD resulted in a smaller increase in energy intake at the end of intervention compared to the first week compared to the NDC and HIIT+ND (Figure 7b).

### 3.5 Absolute and relative handgrip strength

**FIGURE 8:**
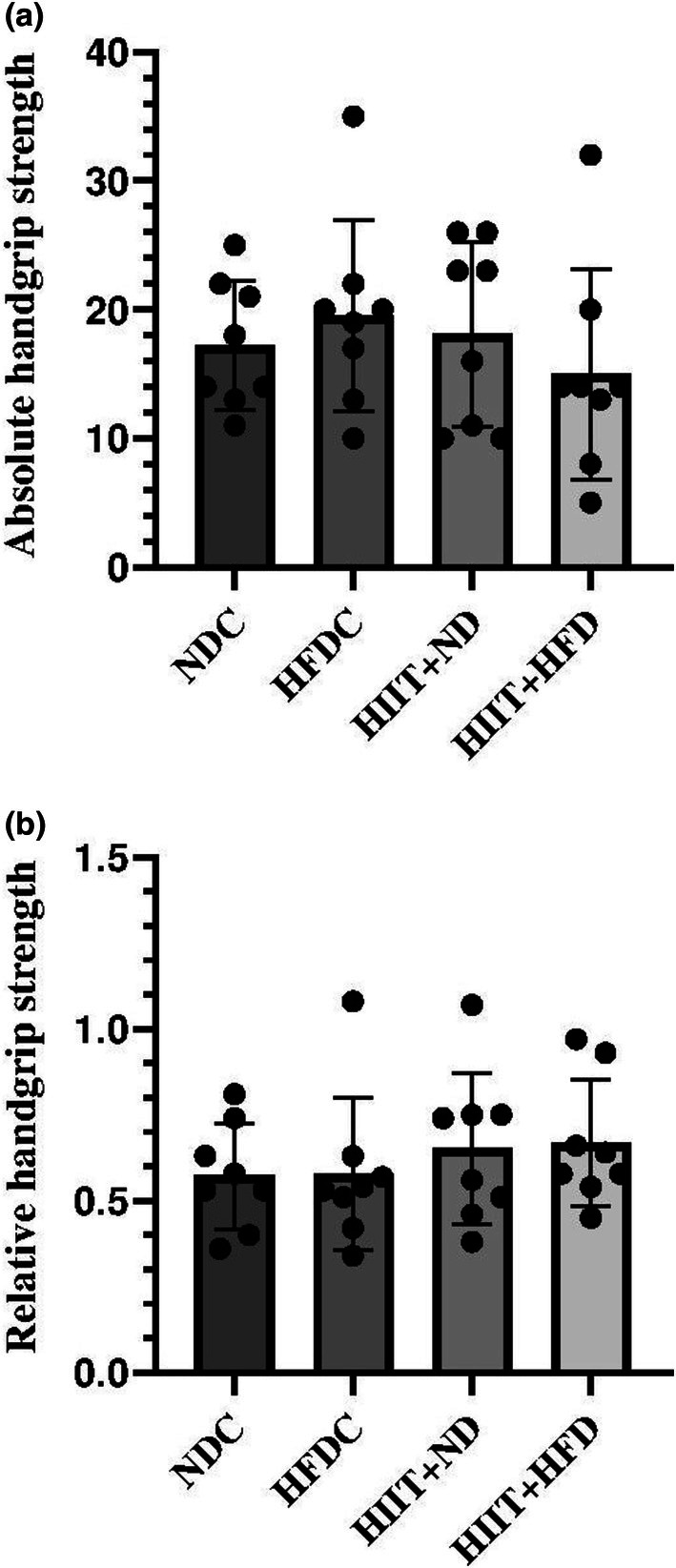
Changes in absolute handgrip strength (a) and relative handgrip strength (b) following 12 weeks of intervention; HFD, High‐fat diet; HFDC, High‐fat diet control; HIIT, High‐intensity interval training; ND, Normal diet; NDC, Normal diet control. Data are expressed as means±SD (*n* = 8) in each group.

Based on the results, HIIT, diet, and the interactive effect of HIIT × diet had no significant effect on absolute and relative handgrip strength (*p* > 0.05, Figure 8a,b).

### 3.6 Associations between variables

The results of Pearson's correlation analysis among variables are shown in Table 4. A positive correlation was found between muscle mass and body weight, food intake, absolute handgrip strength, glycine, and fructose 6‐phosphate levels (*p* ≤ 0.05). In contrast, glutamic acid level showed a negative correlation with muscle mass and body weight (*p* ≤ 0.05). A positive association was shown between the levels of beta‐alanine with energy intake, serine, tyrosine, threonine, and glycine. Also, the tyrosine level was positively correlated with body weight, food and energy intake, glycine, serine, and threonine (*p* ≤ 0.05). A positive correlation was observed between glycine with body weight, serine, threonine, lactic acid, and fructose 6‐phosphate, as well as threonine with glutamic acid, serine, and lactic acid levels (*p* ≤ 0.05). In addition, a positive association was shown between the serine with lactic acid level, and also between the absolute handgrip strength with relative handgrip strength (*p* ≤ 0.05).

**TABLE 4:** Correlation between variables.

	Body weight	Food intake	Energy intake	Muscle mass	Beta‐alanine	Glycine	Glutamic acid	Serine	Threonine	Tyrosine	Fructose 6‐phosphate	Lactic acid	Absolute handgrip strength	Relative handgrip strength
	*r*	*r*	*r*	*r*	*r*	*r*	*r*	*r*	*r*	*r*	*r*	*r*	*r*	*r*
	(*p*)	(*p*)	(*p*)	(*p*)	(*p*)	(*p*)	(*p*)	(*p*)	(*p*)	(*p*)	(*p*)	(*p*)	(*p*)	(*p*)
Body weight	1													
Food intake	0.52* (0.002)	1												
Energy intake	0.60* (0.001)	0.32 (0.07)	1											
Muscle mass	0.78* (0.001)	0.35* (0.04)	0.32 (0.07)	1										
Beta‐alanine	0.32 (0.07)	0.19 (0.29)	0.47* (0.006)	0.09 (0.62)	1									
Glycine	0.38* (0.03)	0.15 (0.39)	0.25 (0.15)	0.37* (0.03)	0.56* (0.001)	1								
Glutamic acid	−0.38* (0.03)	−0.23 (0.18)	−0.14 (0.42)	−0.42* (0.01)	0.18 (0.32)	−022 (0.21)	1							
Serine	0.30 (0.09)	0.01 (0.95)	0.37* (0.03)	0.17 (0.33)	0.75* (0.001)	0.50* (0.004)	0.25 (0.15)	1						
Threonine	0.11 (0.52)	−0.93 (0.61)	0.27 (0.12)	0.02 (0.88)	0.71* (0.001)	0.44* (0.01)	0.38* (0.02)	0.88* (0.001)	1					
Tyrosine	0.34* (0.05)	0.57* (0.001)	0.36* (0.03)	0.31 (0.08)	0.61* (0.001)	0.62* (0.007)	−0.06 (0.70)	0.46* (0.007)	0.44* (0.01)	1				
Fructose 6‐phosphate	0.40* (0.02)	0.05 (0.78)	0.26 (0.14)	0.50* (0.003)	0.19 (0.29)	0.38* (0.03)	−0.07 (0.69)	0.49* (0.004)	0.27 (0.12)	0.23 (0.18)	1			
Lactic acid	0.20 (0.27)	−0.33 (0.06)	0.32 (0.06)	0.26 (0.19)	0.31 (0.07)	0.37* (0.03)	−0.22 (0.21)	0.42* (0.01)	0.42* (0.01)	0.06 (0.71)	0.31 (0.79)	1		
Absolute handgrip strength	0.23 (0.20)	0.24 (0.18)	−0.09 (0.62)	0.34* (0.05)	−0.11 (0.51)	0.12 (0.50)	−0.25 (0.15)	−0.14 (0.41)	−0.14 (0.42)	−0.05 (0.75)	0.07 (0.68)	−0.05 (0.78)	1	
Relative handgrip strength	−0.29 (0.10)	−0.10 (0.55)	−0.09 (0.62)	−0.07 (0.66)	−0.25 (0.16)	−0.11 (0.53)	0.10 (0.55)	−0.19 (0.29)	−0.06 (0.73)	−0.25 (0.15)	−0.09 (0.62)	−0.10 (0.58)	0.81* (0.001)	1

*Note*: r, Pearson correlation coefficient.

* Significant correlation (*p* ≤ 0.05).

## 4 DISCUSSION

This study provides novel evidence that HIIT has more favorable metabolic adaptations in skeletal muscle than diet in male Wistar rats. Our findings demonstrate that HIIT reduces energy intake regardless of diet. Also, HIIT had a significant reducing effect on gastrocnemius muscle mass, beta‐alanine, glycine, serine, tyrosine, and fructose 6‐phosphate, and a significant increasing effect on glutamic acid. The threonine level in the HIIT+ND group was significantly lower compared to both the NDC and HFDC groups. During the study, food and energy intake increased less in the HFDC and HIIT+HFD groups than in the NDC and HIIT+ND groups. These findings provide critical insights into the mechanistic interactions between HIIT protocols and nutritional strategies in governing skeletal muscle metabolic adaptations and systemic metabolic homeostasis. The HIIT with ND had a significantly lowering effect on lactic acid levels.

### 4.1 Mechanistic insights: Metabolite shifts and muscle plasticity

The metabolomic alterations observed in this study are consistent with the known metabolic plasticity of skeletal muscle in response to environmental stimuli (Lahiri et al., 2019). The observed lower levels of glycine, serine, and threonine in the HIIT groups are interpreted as reflecting increased utilization of these amino acids in key energy‐related pathways, rather than a pathological deficiency. These amino acids serve as critical substrates in one‐carbon metabolism and nucleotide biosynthesis processes that support muscle protein turnover, cellular repair, and tissue remodeling (Mäntyselkä et al., 2024; Ramos‐Jiménez et al., 2024). It should be acknowledged that muscle mass measurements in this study were confined to the wet weight of the dissected gastrocnemius muscle. As such, the observed changes reflect local adaptations in this muscle and cannot be generalized to the whole muscle mass. The findings of the present study demonstrate that HIIT significantly reduced muscle mass. This response is likely attributable to the high energetic and metabolic demands of HIIT. Thus, lower steady‐state free amino acid levels can be interpreted as indicative of increased metabolic activity.

The decrease in beta‐alanine in HIIT groups may indicate increased utilization of this amino acid for carnosine synthesis, which is known to buffer intramuscular pH during intense exercise. Carnosine, which is increased with beta‐alanine, attenuates exercise‐induced acidosis through intracellular pH buffering during anaerobic metabolism (Carvalho et al., 2018; Raizel et al., 2019; Verity et al., 2024). Since intramuscular pH was not assessed and metabolite concentrations were measured 48 h after the last training session, the observed reduction in beta‐alanine is unlikely to represent acute exercise‐induced depletion. However, repeated beta‐alanine depletion following consecutive exercise sessions, likely due to cumulative buffering demand, may lead to lower resting beta‐alanine levels over time. Accordingly, decreased beta‐alanine levels most likely reflect chronic exercise‐induced metabolic adaptations related to carnosine turnover and buffering capacity rather than transient post‐exercise utilization. Similarly, reductions in tyrosine levels in the HIIT groups indicate metabolic adaptations such as modulation of catecholamine metabolism, which are linked to improved exercise performance and muscle function (Çınar et al., 2025; Smith‐Ryan et al., 2020). Moreover, elevated ammonia production can stimulate an increase in glutamic acid levels (Fan et al., 2021). Ammonia is recognized as a fatigue‐inducing metabolite in skeletal muscle, particularly under high‐intensity exercise conditions (Holeček, 2022). Therefore, the higher glutamic acid concentrations observed in the HIIT groups in this study may reflect an adaptive physiological response aimed at facilitating ammonia reduction. Trained rats may sustain elevated glutamic acid levels by enhancing its derivation from protein catabolism, a process that likely contributes to the reduction of ammonia accumulation. Our results demonstrate that HIIT, rather than dietary lipid load, dictates the amino‐acidomic signature of skeletal muscle. Twelve weeks of HIIT imposed a dominant metabolic stimulus that reprogrammed intramuscular amino acid turnover, independent of HFD exposure. The absence of HIIT × diet interactions indicates that chronic HFD consumption does not blunt HIIT‐induced remodeling of amino acid metabolism. Notably, the observed decreases in specific amino acids and metabolites reflect enhanced metabolic flux rates that support muscle remodeling, improved bioenergetics, and adaptive responses to exercise training.

Lack of change in isoleucine, valine, lysine, methionine, and phenylalanine levels after HIIT in our study may be related to relatively modest dietary protein intake. Although animals were fed ad libitum and received a diet providing approximately 17–20% of total energy from protein, this level may not have been sufficient to support the increased protein turnover and amino acid utilization associated with HIIT. Regular exercise, particularly HIIT, is generally associated with elevated protein requirements that may exceed the standard recommended daily allowance (Forbes et al., 2020). Considering that essential amino acids play a key role in facilitating adaptive responses to HIIT (Hirsch et al., 2021), similar protein intake between the control and HIIT groups may not have been sufficient to fully support the increased metabolic demands induced by HIIT. Because isoleucine, valine, lysine, methionine, and phenylalanine are essential, levels of these amino acids have been linked to higher dietary protein intake (Gunther et al., 2020; Maddaloni et al., 2025). Some evidence suggests that HIIT can reduce appetite and overall energy intake (Oliveira et al., 2024; Wang et al., 2008), leading to reduced protein intake. It should also be noted that potential HIIT‐induced alterations in appetite regulation or appetite‐related hormones were not assessed in the present study, which represents a limitation and may have affected amino acid availability.

The reduction in lactate levels observed in the HIIT+ND group compared with other groups may reflect favorable adaptations in skeletal lipid oxidative metabolism. Because lactate was assessed under resting conditions 48 h after the last training session, this finding is more likely attributable to chronic metabolic adaptations. Such adaptations could improve energy efficiency production and reduce reliance on anaerobic glycolysis (Takeda et al., 2022; Xie et al., 2024). In contrast, the combination of HIIT with an HFD did not alter resting lactate levels. Given the resting nature of the measurements, this observation may reflect differences in basal lactate handling or metabolic flexibility, rather than direct impairment in mitochondrial oxidative capacity. Additionally, the reduction in fructose‐6‐phosphate levels observed in both HIIT groups may reflect modulation of glycolytic flux and cellular energy management induced by HIIT that appears to occur independently of dietary fat intake. Collectively, these findings indicate enhanced lipid oxidative metabolism. A study has shown that 5 weeks of moderate‐intensity training in diabetic mice led to reductions in skeletal muscle metabolites associated with glucose metabolism, including lactate, glucose, mannose, and tricarboxylic acid cycle‐related amino acids such as phenylalanine, alanine, and serine. This suggests enhanced energy utilization efficiency and improved exercise performance (Xiang et al., 2018). We observed no significant effect in the levels of glucose and 3‐phosphoglycerate in all groups. Amino acid metabolism pathways, particularly those involving alanine, which is critical for muscle energy metabolism and inter‐organ glucose homeostasis through the glucose‐alanine cycle, appear to play a central role in these metabolic adaptations (Han et al., 2023; Ye et al., 2020). BCAAs and their intermediate metabolites are involved in fatty acid oxidation, the tricarboxylic acid cycle, and glycolysis (Ye et al., 2020). So, the lack of change in alanine and BCAAs (isoleucine, valine) may affect the lack of change in the metabolic profile of glycolysis (glucose and 3‐phosphoglycerate). Collectively, findings suggest that exercise training increases the capacity of skeletal muscle to use glucose through glycolysis (Evans et al., 2019; Perry et al., 2008). However, muscle glucose metabolism is influenced by various factors, including the HFD and volume and intensity of exercise (Suginohara et al., 2021), which may be effective in the lack of change of the glucose and 3‐phosphoglycerate in the present study.

In this study, no difference in palmitic acid and stearic acid was observed among the groups. Another study investigated the impact of voluntary exercise training on fatty acid profiles in skeletal muscle. Their findings revealed that 8 weeks of running wheel access led to a selective reduction in 10 specific long‐chain fatty acids (LCFA) species (encompassing both essential and branched types) within the plantaris muscle and three of the 25 LCFAs (including myristoleate, palmitoleate, and eicosatrienoate) of the soleus muscle in male rats (Garvey et al., 2015). Intracellular accumulation of fatty acids affects both muscle mass and function (Conte et al., 2019). Fatty acids may contribute to muscle mass decline by impairing amino acid uptake, essential for protein synthesis and muscle maintenance (Lipina & Hundal, 2017). Another study has also shown that 5 weeks of moderate‐intensity exercise training decreased LCFAs (C14 to C18) and uric acid and its precursors (hypoxanthine, nicotinamide adenine dinucleotide, and niacinamide) of the gastrocnemius muscle in diabetic mice. The accumulation of fatty acids in skeletal muscle could be related to the amount of uric acid and its precursors (hypoxanthine, nicotinamide adenine dinucleotide, and niacinamide). A decrease in uric acid and its precursors in skeletal muscle observed after exercise training suggests enhanced clearance of metabolic intermediates and potential protection against fat accumulation, ultimately contributing to improved energy utilization and muscle function (Xiang et al., 2018). However, in the present study, no significant change was observed in the amounts of palmitic acid, stearic acid, hypoxanthine, and nicotinamide. The absence of any alteration in stearic acid and palmitic acid can be attributed to the absence of any changes in BCAAs (isoleucine and valine). Thus, BCAAs catabolism and fatty acid oxidation could interact with each other (Ye et al., 2020). The absence of alteration in stearic acid, palmitic acid, hypoxanthine, and nicotinamide in this study may be attributed to the rigorous nature of the exercise training regimen. Moderate intensity of training enhances fat utilization and attenuates the levels of LCFAs (C14 to C18) in skeletal muscle and clearance of uric acid, a potential marker of lipid accumulation, potentially contributing to improved physical fitness and muscle composition (Xiang et al., 2018).

### 4.2 Food and energy intake: Implications for body composition

Our data showed that HIIT, along with HFD, resulted in a smaller increase in food and energy intake compared to ND alone, and along with HIIT. Long‐term HIIT can influence appetite and may reduce food intake by inducing exercise‐related appetite suppression or enhancing satiety signaling, phenomena that have been previously reported in other studies (Foright et al., 2020; Wang et al., 2008). The results of our study reveal a positive relationship between body weight and both food intake and energy intake. HIIT may suppress the secretion of appetite‐stimulating hormones, reduce appetite, and decrease dietary intake; these effects are not consistently observed. Studies indicate that higher exercise intensity and longer duration might enhance exercise‐induced appetite suppression and influence appetite‐related hormones, although regular exercise may lessen these effects (Chen et al., 2022). In contrast, another study reported that HIIT reduced caloric intake, body adiposity, total body fat, and weight gain in obese rats, along with improvements in physical performance parameters (Oliveira et al., 2024). Another study showed that rats on an HFD consumed more calories when undergoing HIIT compared to non‐trained rats. However, when on a control diet, trained rats ate fewer calories than non‐trained rats, suggesting that appetite regulation responses depend on diet composition (Arbus et al., 2023). Therefore, the effectiveness of HIIT as a weight‐loss strategy may depend on the diet type (HFD vs. normal diet). Overall, the influence of chronic HIIT on food and energy intake appears to be affected by dietary composition (normal vs. high‐fat), the volume and intensity of HIIT, and the methods used to measure food and energy intake.

### 4.3 Absolute and relative strength: High‐precision measurement is required

Despite changes in various metabolites in our study, there were no significant differences in absolute and relative handgrip strength among groups at the end of the intervention. Although exercise capacity, as measured by muscle strength testing, did not change, rats' running speed increased significantly during the HIIT protocol, indicating improved exercise performance. Muscle strength measurement equipment in our study has not been formally validated, and this tool was researcher‐made, which may have affected the results. Existing evidence suggests that when maximum speed increases, one of the parameters that tends to develop is muscle strength (Pietraszewski et al., 2025). At the end of the protocol, the HIIT+HFD group performed slightly better than the HIIT+ND group, indicating that muscle function and adaptation were not impaired by HFD, in agreement with the maximum speed test results. Nevertheless, the precision of the measurement tool and unanticipated stressors may have affected the observed muscle strength outcomes.

### 4.4 Strengths, limitations, and future directions

A major strength of this study is the comprehensive integration of anatomical, metabolic, and behavioral endpoints, providing a holistic view of muscle adaptation to diet and exercise. Using GC–MS analysis allowed for sensitive detection of metabolite shifts, and the focus on the gastrocnemius muscle—a fast‐twitch, atrophy‐prone tissue—enhances the translational relevance to human metabolic disease.

Nevertheless, several limitations should be acknowledged. Muscle mass measurement in this study was limited to the wet weight of the gastrocnemius muscle, which represents local adaptations that cannot be generalized to the entire skeletal musculature. The exclusive use of male rats limits generalizability, given the known sex‐specific hormonal and metabolic regulation responses to HIIT. Though the duration of intervention may be sufficient to elicit measurable adaptations, it precludes assessment of long‐term remodeling or maladaptive trajectories. Integrating transcriptomic and proteomic layers is necessary to resolve underlying mechanisms because metabolomic profiling captures biochemical states but not regulatory dynamics. Interacting factors—including body weight and composition, energy balance, food intake, and housing temperature—likely influenced metabolic outcomes and muscle plasticity to HIIT. Finally, whole‐muscle homogenates may mask fiber‐type–specific responses, underscoring the need for single‐fiber analyses to define the true architecture of HIIT. These concerns could be addressed in future studies.

## 5 CONCLUSIONS

In summary, this study demonstrates that HIIT affects the metabolic profile of the gastrocnemius muscle in male rats, independently of dietary fat intake, leading to reductions in several key skeletal muscle metabolites. These findings indicate that HIIT exerts a potent metabolic stimulus on skeletal muscle, modulating amino acid‐related pathways and glycolytic flux independently of dietary fat intake. However, further studies are necessary to delineate the precise nature and magnitude of these metabolic adaptations and to elucidate their potential impact on exercise training‐induced physiological adaptations.

## AUTHOR CONTRIBUTIONS

Marziyeh Saghebjoo and Mehdi Hedayati designed research and supervised the project; Asiyeh Taji Tabas, Marziyeh Saghebjoo, Mehdi Hedayati, and Reza Ghahremani: conducted the research protocol; Asiyeh Taji Tabas, Marziyeh Saghebjoo, and Mehdi Hedayati analyzed the obtained data; Asiyeh Taji Tabas drafted the manuscript; Marziyeh Saghebjoo and Mehdi Hedayati reviewed and revised the manuscript; All authors read and approved the final version of the manuscript.

## FUNDING INFORMATION

This research did not receive any specific grant from funding agencies in the public, commercial, or not‐for‐profit sectors.

## CONFLICT OF INTEREST STATEMENT

No conflicts of interest, financial or otherwise, are declared by the authors.

## ETHICS STATEMENT

The approval for all experiments was obtained from the Research Ethics Committees of the University of Birjand, Birjand, Iran (Approval ID: IR.BIRJAND.REC.1400.011).

## DISCLAIMERS

The authors have no disclaimers to report.

## Data Availability

The data analyzed during the current study are available from the corresponding author on reasonable request.
